# Periscope of Medical and Surgical Progress

**Published:** 1886-09

**Authors:** 


### Periscope of Medical and Surgical Progress.

---

NOTES ON PRACTICAL MEDICINE. By ARTHUR B. PROWSE, M.D. Lond., F.R.C.S. Eng.; Assistant Physician, Bristol Royal Infirmary.

SEASONAL PREVALENCE OF PNEUMONIA.—Dr. Seibert, of New York, has more than once directed attention to the close relation existing between those meteorological conditions which favour catarrhal affections and the occurrence of acute fibrinous pneumonia. At the present day, when the specific nature of this disease is receiving much confirmation, it is useful to view the question from another side. In his latest contribution to the subject (*Berl. klin. Woch.*, No. 17, 1886), Dr. Siebert brings forward very strong evidence in favour of the above-named connection. He bases his observations on an investigation of 768 cases of primary pneumonia, distributed in the various months as follows: January 71, February 140, March 103, April 73, May 55, June 37, July 26, August 25, September 43, October 62, November 65, December 78. A comparison with the average temperature of these months shows a striking correspondence between the lowness of temperature and prevalence of pneumonia; and a similar correspondence is manifested with respect to rainfall, except in the summer months, when, in spite of great humidity, there was a minimum of pneumonia. It is also shown that the prevalence of bronchial catarrh closely coincided with that of pneumonia. Dr. Seibert, not content with these general monthly returns, has placed side by side the results of daily meteorological observations, and the dates of occurrence of the cases of pneumonia during each month of the year. Taking the month of February, for example, it is remarkable how closely the onset of the attack follows upon days in which there were combined low temperature, excessive humidity, and high winds. The conclusions at which Dr. Seibert arrives are to the effect that the varying prevalence of the disease can be explained by certain meteorological states which favour its occurrence. These conditions are a low and falling temperature, excessive and increasing humidity, and high winds. When any two of these factors concur, pneumonia prevails to a greater extent than when one only is present; whilst if all three are in operation, the disease is exceptionally frequent. The same states of weather favour catarrh, and catarrh predisposes to pneumonia. It is more prevalent in spring than in autumn, probably, because of the greater susceptibility to exposure after residence in hot and ill-ventilated rooms during the winter, and because of the greater humidity of spring. Finally, the author does not wish it to be inferred that these meteorological conditions are the sole factors at work in exciting pneumonia; his opinion rather is that, by exciting catarrh, they render individuals more susceptible to the influence of the virus.—*Medical and Surgical Reporter*, Philadelphia, June 5th, 1886.

Salol.—This is a salicylate of phenol or carbolic acid, possessing antipyretic and antiseptic properties, recently discovered by Prof. von Nencki, of Berne. It is a white powder, rather greasy to the touch, with a faint aromatic odour, but almost tasteless; nearly insoluble in water, but dissolved readily by alcohol. From experiment on animals, it appears that the whole of it is excreted in the form of urate of salicyl and sulpho-phenol. The pancreatic fluid effects this change; and the fact that this occurs in the duodenum rather than in the stomach, accounts for the absence of nausea and other gastric symptoms. At a meeting of the Medico-Pharmaceutical Society of Berne (*La Semaine Médicale*, April 14th, 1886), M. Sahli related his experience of the drug, and said he had taken two drachms in a day without experiencing any disagreeable effects, though some of his patients noticed a slight ringing in the ears after large doses. It sometimes colours the urine almost black, just as carbolic acid does. In rheumatism he had found it act quite as well as salicylic acid. One case of chronic urticaria which had resisted every kind of treatment was quickly relieved, as were also several cases of obstinate suborbital neuralgia. In doses of 30 grains it was a powerful antipyretic, reducing the temperature in a case of phthisis 7° Fahr., without causing any unpleasant symptoms. As an antiseptic, it had yielded good results in ozæna and otorrhœa. Other speakers at the meeting confirmed the above statements as to its value, and united in recommending a further trial in rheumatism, fevers, and septic diseases. (*Med. Record*, New York, June 19th, 1886.) Salol would be indicated also in diabetes, as phenol is given. It is given as a local antiseptic in all intestinal catarrhs, and may be used in typhoid fever, cholera, &c., and against intestinal parasites. As an antiseptic in place of iodoform, it has given such satisfactory results that it is likely to supplant that unpleasantly odorous substance in many ways. The urine of patients taking it becomes completely aseptic; and urine kept in contact with it does not decompose.—*Philadelphia Medical Times*, May 15th, 1886.

Remedy for Hiccough.—Dr. A. G. Gibson, in the *Edinburgh Med. Journ.*, April, 1886, calls attention to the Hippocratic aphorism, "Sneezing occurring after hiccough, removes the hiccough," and says that he has proved its truth by experience. Hiccough, like sneezing, is a modified respiratory movement; and it is quite in accordance with what we know of the transference of nervous action, that the induction of an explosive expiratory act should cause the cessation of the diaphragmatic spasm. Tickling the nostrils, or the application of some gentle irritant, even though it does not cause actual sneezing, is often effectual. In the *Med. Record*, New York, June 5th, 1886, Dr. R. B. Williams recommends the use of a "catarrh snuff," made by mixing one part of powdered veratrum album with four parts of powdered liquorice root. He says: "I have seen the happiest results from it in hiccough of as much as two days' persistence. Of course, it may fail in very stubborn cases; but it never has, thus far, with me."

Catarrhal Jaundice.—The use of cold-water enemata in this affection was first proposed by Krull in the *Berl. klin. Woch.*, p. 159, 1877. He injects two to four pints, at a temperature of 60° Fahr., which is to be retained as long as possible; the enemata being repeated twice a day if necessary. Löwenthal's plan is to use water at 60° Fahr. the first day; on succeeding days an enema is given every morning, the temperature being gradually increased up to 71·5°, which is not exceeded. The cure is generally accomplished by the fourth day; and in no instance have more than seven injections been found necessary. Several cases were of long standing, and had resisted a great variety of treatment. No medicine is given, and a vegetable diet is adhered to.—*New York Med. Journ.* and *Journ. American Med. Assoc.*

Reflex Nasal Cough.—In the *Practitioner* (July, 1886), E. Cresswell Baber, M.B., gives some interesting cases illustrative of this affection. The conclusions he draws are as follows:—(1) Reflex nasal cough is only exceptionally produced by probing the anterior part of the inferior and middle turbinated bodies and the tubercle of the septum.

(2) In children it is not very uncommon to find that a slight hacking cough can be produced by irritating the anterior end of the inferior turbinated body. (3) The cough reaction may occur without erection of the inferior turbinated bodies, and may be intermittent in character. (4) Reflex nasal cough and the act of sneezing are very closely associated, and probably represent different forms or degrees of the same irritation.

The practical outcome of these considerations is, that in cases of spasmodic cough not otherwise to be accounted for, we should do well to examine carefully the nasal cavities in regard to the reflex irritability of their lining membrane.

Sodium Sulphate as an Aperient.—Nöthnagel a year or two ago investigated the action of neutral salts on the intestines. He found that sulphate of sodium—Glauber salt—was the mildest of all; that it first of all caused a slight redness, and, later, induced depletion by emptying the congested capillaries, and produced a slightly watery stool. In the inflammatory intestinal disorders of childhood, if administered early in moderate doses, it acts almost as a specific; while sulphate of magnesia induces a severe congestion, and generally destroys the epithelial lining. Experience has proved this observation to be correct; for, generally, when a large dose of Epsom salt has been given, the patient will suffer from constipation, or at least from sluggishness of the bowels, for several days afterwards.

The action of Glauber salt is so certain, and at the same time it is so much a true laxative,—not injuring the epithelial lining,—that a smaller dose is generally needed than in the case of sulphate of magnesia. In most cases one-half to one teaspoonful, if administered to an adult, largely diluted with water, one hour before breakfast, will induce one or two motions of the bowels about half an hour after the meal; and this result is brought about without the least griping, and without inducing subsequent constipation.—*Med. and Surg. Reporter*, Philadelphia, June 5th, 1886.

[I have prescribed sodium sulphate largely, in place of the magnesium salt, for out-patients of all ages at the Royal Infirmary, and also in private practice, and am convinced of its value as a simple and efficient aperient. It is especially adapted for children, on account of the mildness of its action, and also because it has not a nauseous taste like Epsom salts. As far as I can judge, it deserves to be classed as a "good old remedy," which has undeservedly gone out of fashion at the present time.]

Cocaine, and some of its Uses.—*Sea-sickness.*—Dr. C. F. Mason writes to the *Med. Rec.* (N.Y.), June 19th, 1886: "In a recent trip from New York to Norfolk by sea, I had an opportunity of observing the remarkable curative effects of cocaine in sea-sickness. The cases treated (four in number) were all adults, two being physicians. The symptoms were extreme nausea and great depression; no vomiting. Cocaine hydrochlorate, in doses of twelve minims of a four-per-cent. aqueous solution, afforded almost complete relief in from fifteen to thirty minutes, so that the patients were enabled to dress and come on deck. After a second half-grain dose (two hours' interval) the relief was complete and permanent."

*Neuralgia.*—Dr. de Coninck (*Le Moniteur du Practicien*, Jan. 15th, 1886) states that in cephalalgia, having its seat in the temporal region, cocaine hydrochlorate, in one-per-cent. solution, has remarkable effects. The pain, however intense, will cease at once on applying to the external auditory canal a minim of the solution. A second application may be required after a few hours. In neuralgia of the fifth nerve and its branches the results were less certain, owing, perhaps, to the superficial mode of its employment.—*London Med. Record*, March, 1886.

*Laryngeal Ulceration.*—The pain and distress resulting from ulcerations within and about the glottis in advanced stages of tubercular phthisis can be greatly relieved by spraying or mopping the parts with a four-per-cent. solution of cocaine, or by placing a cocaine tablet on the base of the tongue. Phonation becomes more perfect and less laboured, respiration less difficult, and deglutition comparatively easy. The relief lasts four or five hours.

*Hay-Fever.*—Dr. E. F. Ingalls of Chicago and Dr. J. M. Costa have found it very valuable in this affection; and the latter uses it also in rose-cold. The strength used is from four to eight per cent., applied as a spray or by means of a dropping-tube. There are, however, persons who seem to be wholly insusceptible to its influence.

*Pruritus Vulvæ* is usually greatly relieved by the use of a four-per-cent. solution; and the same may be said of local irritations in the neighbourhood of the other mucous orifices of the body.

*Earache.*—Dr. S. Bishop of Chicago finds that it gives prompt relief, which is often permanent, even in cases of actual internal otitis. He prefers the drug in solution, rather than as a powder.—Dr. A. H. Agard, in the *Pacific Med. and Surg. Journ.*, June, 1886.

TREATMENT OF HYDATID DISEASE.—An interesting paper on this subject was recently read by Dr. W. Gardner before the South Australian Branch of the British Medical Association. It dealt principally with pulmonary hydatids. Taking all the statistics at command, it seems that of 212 cases of spontaneous rupture, 101 recovered and 111 died—less than fifty per cent. of recoveries. Bruen, in his article on "Pulmonary Hydatids," in Pepper's *System of Medicine*, says that thirty or forty per cent. of cases terminate in recovery if the cysts burst spontaneously; death being caused in others by suppuration and exhaustion. Dr. Gardner's conclusion is, that the expectant method will only be applied in the case of ruptured cysts which are causing little inconvenience and are in process of cure, and such cysts as are so situated as not to be amenable to surgical treatment. He holds that all hydatid cysts of the external surface of the body should, immediately upon the establishment of the diagnosis by the aspirator or hypodermic needle, be incised, and the cyst removed; and that hydatids of the lung or abdomen should be first tapped, in the hope of thus effecting a cure. A radical operation should be resorted to on the appearance of suppuration or of opacity of the fluid.—*Journ. American Med. Assoc.*, June 5th, 1886.

Dr. Spisharny, of Moscow, describes, in *Vratch*, three cases of hydatid disease of the liver under the care of Professor Sklifassovsky; all terminating in recovery. The Professor is strongly in favour of free incision, and sewing of the wall of the cyst to the edges of the abdominal wound. Statistics are in favour of this proceeding. Neisser has collected 300 cases of hydatid disease of the liver treated by different methods with Listerian precautions. The results were mostly unsatisfactory. The majority of this series were treated by puncture. In another series of 132 cases treated in Mecklenburgh, the cyst in twenty-five burst spontaneously; fifty-two per cent. of these died. In seventeen the cyst was punctured; the mortality being fifty-eight per cent. In twenty-eight cases incision and fixation of the cyst-wall was performed, with only twenty per cent. mortality. Korach has employed incision in eighteen cases; in six of these the incision through integuments and cyst-wall was done in two stages. All of the latter recovered; one of the remaining twelve died. Incision is becoming the most popular method amongst Russian surgeons, who have considerable opportunities of studying hydatid disease in their wards. There can be no doubt that aspiration is a dangerous proceeding. Incision is generally satisfactory, provided that there is no cyst behind that which is opened. If there be others, the risk of fatal suppuration will be very great.—*Journal American Med. Assoc.*, July 3rd, 1886.

Substitutes for Fehling's Solution.—Prof. Holland gives the following as a test for sugar; it is very efficient, easily prepared, and is not spoiled by keeping: Dissolve ʒi of cupric sulphate in ℥i of glycerine. Add 5 drops of this to ʒi of liq. potassæ in a test-tube. Boil a few minutes, to test its purity. Should it remain clear, add a few drops of urine. If glucose be present in quantity, the red precipitate is at once formed. To detect minute amounts of sugar, ʒss of urine should be added, and the fluid again boiled. As it cools it will turn an olive-green colour, and become turbid, even if a very minute quantity of glucose be present.—*Canadian Practitioner.*

M. Schmiedeberg uses mannite in place of Rochelle salt in making Fehling's solution. He says it ensures stability to the solution. The formula is as follows: Dissolve 34·632 grammes of crystallised copper sulphate in 200 cc. of water; to this solution add a solution of 15 grammes of very pure mannite in 100 cc. of water, and 480 cc. of solution of caustic soda (1·145 sp. grav.); and, lastly, sufficient water to make one litre.—*American Journ. of Pharmacy*, Jan., 1886, and *Practitioner*, June, 1886.

Action of Potassium Bichromate.—Dr. Alfred Drysdale publishes in the *Medical Press*, April 21st and 28th, 1886, an elaborate paper on the sphere of action of this salt. After describing its very marked and characteristic effects in those cases in which it is rapidly fatal as well as in chronic poisoning, he suggests that as a remedy it may be reasonably hoped to modify pathological processes in tissues which are specifically acted upon by it, such as the respiratory passages and alimentary canal, the liver, kidneys, skin, and bones. It has been used, and with a certain amount of success, in scrofulous eczema, farcy, digestive disorders, marasmus, and syphilis. Polypi of the nose, recurrent after repeated removal with the forceps, will often yield to the topical application of a snuff composed of one part of potassium bichromate to nine parts of sugar of milk. It also promises success in the treatment of hay-fever; and, as a gargle in acute ulcerated sore throat, as well as in cases of indolent enlargement of the tonsils (2 grains to ℥i of water with ℥ss of glycerine). When given internally, as in the treatment of certain forms of dyspepsia, chronic intestinal catarrh, and in syphilis, the dose should not be more than $\frac{1}{4}$ grain daily, though sometimes $\frac{1}{5}$ grain will produce violent emesis.—*Therapeutic Gazette*, June, 1886.

Pertussis.—The stubborn resistance this affection offers to therapeutic skill is clearly shown by the constant references to its treatment in medical journals all over the world. So many vaunted remedies prove valueless in the light of further experience, that one hesitates to call attention to any new plan of treatment. Still, among the chaff, there must be some few grains, therefore the following statements are given for trial in the sieve of practical experience:

(1) Dr. Moncorvo, of Rio de Janeiro, recommends painting the isthmus of the fauces and the laryngeal orifice with a solution of cocaine (10 per cent.); to be followed in a few minutes with a solution of resorcin, which he believes is the specific germicide of the disease.—*London Medical Record*, March 15th, 1886.

(2) At a recent meeting of the Société de Biologie, MM. Brown-Séquard, Laborde, and d'Arsonval, united in praising the effects of narceine in whooping-cough and chronic bronchitis with hypersecretion. This alkaloid exists in opium, but in very minute quantities—about ·02 per cent.—and its formula is $C_3\ H_{29}\ NO_9$. Most investigators conclude that in its action upon man it resembles morphine, but is milder. The testimony heretofore has been so conflicting that doubts have arisen as to whether the same alkaloid has been used. M. Laborde now says that a very easy test of its purity is to add to the solution a drop of hydrochloric acid, when a beautiful blue colour is produced. The usual dose for an adult is $\frac{1}{2}$ to 1 grain; and for a child, $\frac{1}{6}$ grain dissolved in syrup.—*Medical Record*, N.Y., July 17th 1886.

[The first notice of its value in pertussis occurs in the *British and Foreign Medico-Chirurgical Review*, 1866.]

(3) Dr. W. T. Parker, of Newport, Rhode Island, advocates the use of quinine, in doses of $\frac{1}{2}$ to 1 grain, or more, every two hours, for even young children; and says that improvement begins after the first two or three doses. Dr. Dawson, also, employs small and repeated doses. Drs. Sauerhering and Bachem use insufflation of a powder of quinine hydrochlorate and gum arabic (3 to 1).—*Philadelphia Medical Times*, July 26th, 1886, and *Medical Record* (N.Y.), July 3rd, 1886.

(4) Dr. W. T. Greene (*Medical Press*, April 7th, 1886; *Therapeutic Gazette*, May, 1886) suggests an improvement on the old plan of sending children on visits to gas-works. He attaches a piece of rubber tubing to a burner, the tubing being long enough to reach the floor. The gas is then turned on just enough to make its odour perceptible, and the child allowed to stand over and inhale it for a few minutes, several times a day. After a few inhalations, great improvement is said to result.

(5) A Tasmanian correspondent of the *Australasian Medical Gazette*, May, 1886, recommends the use of carbolic acid internally, but does not state the dose he gives.

[After a somewhat extended trial of many drugs in whooping-cough, I have come to the conclusion that carbolic acid is more frequently efficacious than any other in controlling the cough and sickness. One-sixth to one-third of a minim (increased cautiously, if necessary), in combination with simple syrup, ʒss to ʒi, forms a mixture which is not unpalatable to most children, and this may be given every five or six hours.]

Treatment of the Hysterical Attack.—Dr. Albert Ruault gives a simple method which he has found very efficacious in controlling the hysterical fit. It consists in maintaining firm pressure over the supraorbital nerves at the points of emergence from the foramina. The head is held securely between the palms of the hands, while pressure is made on each side with the thumbs. He says that the patients under this treatment first contract the facial muscles with an expression of pain, cry out, and then take several quick successive inspirations. The breath is held for a few seconds, and then, with a long expiration, the muscles relax and the attack is ended. The pressure should now be relaxed, otherwise it may have the opposite effect, and excite another convulsion. Pressure over any nerve-trunk at the point where it becomes superficial will have the same effect; but the supraorbital nerves are chosen because of their convenient situation.—*France Médicale*, and *American Pract. and News*, July 10th, 1886.

Infant Feeding.—Dr. A. V. Meigs, in the *Philadelphia Medical Times*, June 12th, 1886, says that for a new-born child the best artificial food is the following: Two tablespoonsful of cream, one tablespoonful of milk, two tablespoonsful of lime-water, and three tablespoonsful of sugar-water; the latter being made by dissolving $17\frac{3}{4}$ drachms of sugar of milk in a pint of water. For older children Mellin's food may be added in the proportion of one level teaspoonful or more to the ounce of food. He objects to the use of cow's milk, either pure, or diluted and sweetened.

How to Prevent Colds.—Dr. Brown-Séquard, speaking at the Societé de Biologie, said: "The neck is one of the most sensitive regions of the body, and it is not doubted by anyone that a large number of cases of bronchitis, &c., are reflex phenomena from the impression of cold on the nerves of the skin in that region. To prevent these troubles, the sensibility to cold must be diminished. To this end I have advised several of my patients to blow air on the neck from a blower, at first using warm air and gradually cooling it off. In ten sittings most of them had become so accustomed to it that they became quite impregnable to the action of cold." In America, water is used instead of air for the same purpose.—*American Pract. and News*, June 12th, 1886.

Menthol in Urticaria and Pruritus.—Among the myriad of remedies for these troublesome affections we have no other which affords such complete and instantaneous relief as a solution of menthol. We have used this remedy for urticaria in three cases. Not only is the itching relieved for the time, but a cure seems to be effected. In pruritus ani and in eczema, moistening the parts with menthol solution causes an immediate cessation of pain. The solution should contain from 2 to 10 grains of menthol to the ounce of water.—*Pharmaceutical Journal*, May, 1886.

Sciatica—Puncture of the Nerve-sheath.—Sir Joseph Fayrer states, in the *Practitioner*, April, 1886, that some years ago he was asked to see a case of severe sciatica of long standing in a man of middle age. The pain was very severe, continuous, liable to increase, and of a paroxysmal character. The posterior muscles of the thigh were somewhat atrophied. The patient himself was wasted and worn by continued suffering and deprivation of rest and sleep. There was no history of rheumatism, gout, syphilis, or other specific cause. A malarial origin, of course, was possible; but there was no satisfactory explanation of the origin of the disease. All the usual methods of treatment had been resorted to, but without relief. On examining the limb, he detected some fulness and tenderness with slight fluctuation in the course of the sciatic nerve at the upper part of the thigh. He, therefore, introduced a long narrow knife into the swelling until it entered the sheath of the sciatic nerve. This gave exit to about two drachms of clear, serous fluid: immediate relief followed, and ultimately complete recovery. He says that he has seen other cases—none, however, so well marked as this one—where puncture has given relief; but he is not aware of others having similar experience, and would therefore call attention to it as of practical interest.

Germicides, so-called.—Klein, in a recent publication, claims to have demonstrated that many of the most popular antiseptics, as now used, are as harmless to the life of the microbe as water. The matter is one of great practical importance. Unless his conclusions are disproved, they will weaken confidence in all germicidal measures. He has found that quite weak solutions may render germs quiescent, even for long periods; but this is no proof that they are lifeless, for when they are subsequently placed in media suitable to their development, they become active, and increase rapidly. He has exposed for twenty-four hours spores of the bacillus anthracis to Calvert's fluid, pure terebene, phenol (10 per cent.), and mercuric chloride (1 per cent.), and then inoculated guinea-pigs with them, using spores and antiseptic and all in the process. The animals died of anthrax, and the blood was found teeming with the bacillus.—*Pacific Med. and Surg. Journal*, June, 1886.

---

Note on Hemi-Rheumatism, or the one-sided predominance of manifestations of Chronic Rheumatism.—M. Cazalis draws attention to a feature of chronic rheumatism which he and others, as well as Dr. Demeaux, have frequently observed, but which has not been described in any of our classical treatises. The conclusions of his work are as follows:—

(1) In more than two-thirds of chronic rheumatics there exists, in some cases during a long period, a tendency to arthritic manifestations, external or internal, predominating on one side of the body; and sometimes this predominance is such that these cases might be called hemi-rheumatics.

(2) In these hemi-rheumatics the right side appears the most frequently affected.

(3) When pulmonary congestion, or bronchitis of an arthritic nature, shows itself in these patients, these conditions oftener appear on the side in which the rheumatism preponderates. It is on that side is observed that pleuritic friction which M. Collin (de Saint-Honoré) has considered to be an indication of arthritism, and which, with M. Cazalis, corresponds to the friction or crepitation of the joints in dry arthritis. As hemi-rheumatism is oftener observed on the right side, one understands why M. Collin at once localised his pleuritic friction on the right side.

(4) On the side on which rheumatism predominates, is also more frequently observed that other and more constant sign of chronic rheumatism—sometime ago recognised and described by M. Verneuil and M. Potain—the deformity and projection of the great toe.

(5) If, in simple chronic rheumatism, hemi-rheumatism appears oftener to affect the right side, hemichorea and hysteria, on the contrary, which to many practitioners of the present day seem to be transformations of arthritism from one generation to another, more frequently affect the left side. Now, if hemicrania is added to these arthritic attributes, a singular tendency to hemilaterality will be recognised in all these arthritic modifications.

(6) These facts seem to support the still uncertain theory, but which is becoming more and more in favour, that the central nervous system greatly influences the localisation of these chronic rheumatic manifestations.—*Bulletin de l'Académie de Médecine*, No. 17. April 27th, 1886.

R. Shingleton Smith.

---

### OPHTHALMOLOGICAL NOTES.

By F. Richardson Cross, M.B. Lond., F.R.C.S.; Surgeon to the Bristol Eye Hospital; Ophthalmic Surgeon to the Bristol Royal Infirmary.

The Relation between Dental and Ocular Affections.—In his presidential address to the Bath and Bristol Branch of the British Medical Association, Mr. Gaine described a case of ptosis and amaurosis, associated with fractured upper molar and pus in the antrum. Removal of the stump and evacuation of the pus was followed by disappearance of the ptosis.

Dr. Ward Cousins showed, at Brighton, an incisor tooth, perfect in outline and in structure, which he had removed from the orbit of a child two years old. Mr. Tracy regarded this as a tooth displaced during an early stage of development.

Burnett *(Archives of Ophthalmology)* reports a case of abscess in the superior maxilla and orbit, followed by atrophy of the optic nerve, which resulted from an unsuccessful attempt to extract the upper canine tooth.

At the recent Ophthalmological Congress, held in Paris, M. Paul Redard described a number of cases in which dental affections were evidently the source of ocular disturbance, such as glaucoma, amblyopia, and cloudy vision. In asthenopia without any apparent cause, the teeth should always be examined. M. Gayet mentioned a case in which ocular disturbance was produced by a tooth fixed on a pivot. The symptoms appeared and disappeared according as the tooth was removed or replaced. M. Fieuzal had observed so many of these cases of correlation between ocular and dental affections, that he had urged that a dental clinic should be annexed to the Quinze Vingts Hospital for blind people. M. Suarez and M. Galezowski mentioned similar facts. M. Gaval mentioned a series of cases, of an inverse order, in which dental disturbance disappeared after operating for glaucoma.—*Brit. Med. Journ.*, June 19th, 1886.

Glaucoma.—M. Panas, in a paper read at the Académie de Médecine, in June, 1886, concludes (1) that, in certain forms of glaucoma, myotics act not only as palliatives, but as curative agents; (2) to obtain the best results from myotics, their use must be extended over a considerable period of time; (3) that after operation, or in cases where operation seems unadvisable, myotics are effective in preventing the progress of the disease.

*Glaucoma caused by Instillation of Cocaine.*—(1) Maier relates a case of a man, 52, who, having lost one eye at least ten years previously by absolute glaucoma, came one evening with slight symptoms of glaucoma in the other eye. Eserine was ordered, pending a sclerotomy. On the following morning, a condition of acute glaucoma had developed, and this was attributed by Maier to the instillation of one drop of cocaine in the evening and another in the morning, in mistake for the eserine. (*Lond. Med. Rec.*, June, 1886.) (2) Manz also reports a case of a woman, aged 50, with presbyopia, who came to him for glasses. Distant vision was normal; the anterior chamber was shallow, and there was a deep physiological cupping of the disc. Cocaine was used in the right eye, to facilitate examination. On the following day, Manz was sent for, to find in this eye a typical attack of acute glaucoma; he was told that severe pain in the eye, and headache, had come on the previous evening, with loss of sight. Iridectomy was at once performed, with a good result.—Abstract in *Inter. Journ. of the Med. Sciences*, April, 1886.

Prof. Guaita, on the contrary, considers that cocaine is very valuable in secondary and hæmorrhagic glaucoma; that it reduces ocular tension, and produces relaxation of the muscles within and connected with the eye.

The Cause of Regular Astigmatism.—At the Kentucky State Medical Society, held at Winchester, in June, 1886,

Dr. Allen Kelch, of Louisville, read a paper on this subject. He excluded (*a*) the aqueous humour and (*b*) the vitreous from being possible seats of the defect. Astigmatism shows a constant invariable amount of error under all conditions of the eye-focus; whatever the distance of the object looked at, it is the same cylinder which is always required, and that in a constant position. (*c*) The lens, which undoubtedly is the structure concerned in altering the focus of the eye in accommodation, cannot be concerned in astigmatism. Failure of accommodation is aided by spherical lenses, and not at all by cylinders, unless these are also required for distant vision. (*d*) The cornea still remains for discussion. The pressure of the lids upon the globe tends to compress the cornea in the vertical meridian, and to lengthen it in the horizontal, by which the refractive power is increased in the vertical and diminished in the horizontal meridian respectively. In order to neutralise these defects, cylinder glasses must be placed so as to increase the refraction of the horizontal meridian, or to lessen that of the vertical; that is, a convex cylinder must be put with its axis vertical, or a concave cylinder with its axis horizontal. Statistics of 900 cases are quoted, in 90 per cent. of which it was necessary to adjust the cylinders as above indicated. Dr. Kelch concludes that (1) regular astigmatism is due to changes in the spherical form of the cornea; (2) the cornea has its contour altered by the pressure of the lids, and in this pressure we find the cause of regular astigmatism.

[The image thrown upon the cornea by means of various instruments is shaped according to the corneal curvature, and this special shape of the image shows a constant relation to the astigmatic defect. Iridectomy and external operations also cause astigmatism. My experience of the necessary position of the cylinder agrees with the statistics quoted by Dr. Kelch; but a tilt of a few degrees is frequently necessary.]

Retinitis Pigmentosa.—In the American Ophthalmological Society, July, 1886, a discussion on the "Possible Retardation of the Progress of Retinitis Pigmentosa" was opened by Dr. Derby, of Boston. Several of the speakers related slight but definite improvement under use of the constant current. Absolute rest of the eyes was considered of great importance; but a case was quoted where very little change had occurred during eight years in a student who was twenty-four years old when the disease had been first diagnosed. The only drugs that seem of special service are mercury and iodide of potassium, where a syphilitic constitution may be suspected.—*Medical Record*, New York.

